# A Treatise on the Etiology, Pathology, and Treatment of Congenital Dislocations of the Head of the Femur

**Published:** 1850-07

**Authors:** 


ART. VII.—*A Treatise on the Etiology, Pathology, and Treatment of Congenital Dislocations of the Head of the Femur. Illustrated with Plates.* By JOHN MURRAY CARNOCHAN, M. D. Lecturer on Operative Surgery with Surgical and Pathological Anatomy. New York: S. S. & W. Wood.

This treatise is devoted to a species of disease that is very rare in this country. We have neither seen a case of it, nor have we ever met with a physician who referred to it. But the rarity of the disease is an excellent reason for studying this book thoroughly, for although little hope may be entertained of a cure, a full knowledge of the subject may prevent a physician from doing harm. This is surely a matter of some interest to the practitioner of medicine, and of advantage to the patient. Palleta, an Italian Surgion, was the first who gave a distinct account of congenital dislocation of the femur. He published his observations in 1820. In 1826 Dupuytren, without noticing Palleta's publication, read before the Academy of Sciences, an essay entitled: "*Mémoire sur le Déplacement Originel on Congénital de la Téte du Fémur.*" This excited a great deal of attention in Paris, at the time of its publication. Since that time the subject has been elucidated by Breschet, Sedillot, Pravas, and particularly by Jules Guérin.

Dupuytren gives a history of a family, in whose members there was an extraordinary tendency to this disease. We quote this curious detail: "There exists in the town of Nantes, a family, many members of which have been, and are, affected with original luxation (*luxation originelle*) of the femurs. The oldest member of this family is a woman eighty years of age, named *Marguerite Gardas*, fruiterer, and here follow the particulars of her case, taken from her own lips, and corroborated by other persons of the same age:

" 'Two of her aunts, on the maternal side, who died at seventy years of age, had been affected with a lameness from their earliest attempts at walking. These females, moreover, were in the habit of stating that they had always been lame. Their hips were elevated, protuberant, and abruptly salient. They walked with their elbows projecting backward, and hobbled like ducks. Marguerite's father had had a sister lame upon the right side from birth, who died at eighty years of age. Another sister, who was well formed, gave birth to a female child, which presented a shortening of the right inferior extremity.

" 'Marguerite Gardas, who is the subject of this observation, is a large and robust woman, and appears to have possessed a remarkable degree of activity in her youth. With her the disease was not prominently marked until she was thirty years of age, and its symptoms are those of a spontaneous luxation* (*luxation spontanée*) of the femur. The affected member is one fourth less in diameter than the other, which is, besides, three or four lines longer. This woman has had, by her marriage with a healthy man, who had come from distant parts, a daughter named *Simone*, who has a congenital shortening of the right inferior member of about three inches. This girl is also married to a man of healthy conformation, but whose father had a congenital luxation of both femurs; she has had four children, two of whom present the hereditary infirmity. The one is a young woman aged twenty-three years; she has a luxation of the two femurs, the heads of which are situated in the *external iliac fossæ;* the other is a young man twenty-one years of age, who has a congenital luxation of the left thigh. The member affected is shorter by five inches than the other; the head of the femur has ascended upwards and backwards; the trochanter major projects forwards and outwards, and the toes are turned inwards; the two extremities are equally well developed.' "

The anatomical observations are very interesting, but we must pass over them, and present the etiology of the affection to our readers. Dr. Carnochan says: "The congenital dislocations of the head of the femur occurring at the ilio-femoral articulation, are either *complete* or *incomplete;* and they may exist upon one side only, or, as

---

*The word *spontanée,* in the French text, is here used as synonymous with *originelle.*

most frequently happens, the displacement may exist simultaneously on both sides, forming a double luxation.

"The head of the femur may be completely dislocated before birth in three different directions:

"*Firstly*, upwards and outwards; this form of dislocation corresponds to that variety which is described by authors as being situated upon the dorsum of the ilium. It is the variety which is most common, and to it practical authors have chiefly directed their attention. The head of the femur is here situated, in the new-born child, above the margin of the acetabulum, and, in the progress of time, mounts upon the dorsum of the ilium, and rests in the *external iliac fossa*. Of this variety I have seen many examples, and the accompanying plates, taken from an adult during life, and also from some dissections I have made, will illustrate the appearance and nature of the luxation in this direction. This form is generally a double luxation, but it is also met with upon one side only.

"*Secondly*, the luxation directly upwards. This kind of dislocation has been seen only in those fetal monstrosities which have been called *agénosome*, in which, with other anomalies, the abdominal walls are not completely developed. The head of the femur is here placed immediately external to the anterior and inferior spinous process of the ilium.

"*Thirdly*, the luxation forwards and upwards. Like the preceding, this displacement has been only met with in the fetal monstrosity. The head of the bone rests upon the *eminentia ilio-pectinea*, and forms a well marked tumor in the groin.

"M. Guérin mentions in addition to the forms already noticed, a sub-luxation upwards and backwards, which in reality appears to be but a modification or incomplete development of the dislocation upwards and outwards.* He describes it as being characterized by the partial escape of the head of the femur, which does not pass beyond the margin of the acetabulum. This variety is met with in new-born children, and sometimes in cases where luxation from muscular *retraction* has occurred spontaneously soon after birth.

"The same author has also indicated two varieties of what he terms *pseudo-luxations* of the hip; one, simulating a luxation backwards and outwards; the other, resembling a luxation downwards and forwards. 'There exists,' he says, 'an order of congenital dislocations of the hip, to which I have given the name of *pseudo-luxations*, because they present the fallacious appearance of luxations, although the head of the femur has not escaped from the cotyloid cavity; the varieties of these pseudo-luxations are themselves the result of the muscular retraction, differently distributed among the pelvi-femoral muscles.'

"This pathologic condition of the pelvi-femoral muscles had already been noticed by Delpech,† and the novelty presented by M. Guérin consists in the adoption of a designating term, which serves a useful purpose in individualizing a class of affections.

"From this enumeration of the varieties of the congenital dislocations occurring at the hip-joint, it will be seen that several of them are only to be met with in the imperfectly developed fetus, and consequently cannot come within the range of surgical practice: others, however, are met with in the living individual, and become the object of remedial attention. The former are interesting in

---

*Strictly speaking, the head of the femur, in the dislocation upon the dorsum of the ilium, passes upwards and backwards. I have retained, however, the designation adopted by Dupuytren, "*en haut et en dehors*."

†De l'Orthomorphie par rapport à l'espèce humaine.

a scientific point of view only; the latter belong to the practical details of the profession.

"To no other class of maladies is the phrase, '*felix qui potuit rerum cognoscere causas*,' more applicable than to the congenital dislocations observed at the ilio-femoral articulation.

"The researches into the origin and nature of the class of displacements which occur at the articulations of the fetus, while still in the womb of the mother, have perhaps suggested more ingenious hypotheses than any other of the obscure parts of surgical pathology. Laying aside the predisposition engendered by hereditary transmission, and by sex, as being generally concurred in, the other causes mentioned by writers, as tending to bring about the dislocation in question, may be referred to different heads. First, external violence, acting upon the fetus while in utero *(J. L. Petit;)* second, a primitive alteration in the germ, or an aberration of the formative power *(force formatrice) (Dupuytren;)* third, an arrest in the development of the osseous portions forming the cotyloid cavity *(Breschet;)* fourth, certain articular maladies, occurring in the fetus during intra-uterine life *(revived by M. Parise and others;)* fifth, a primitive alteration in the nervous centres *(Chaussier,)* revived by Delpech and Guérin; and lastly, to be more definite, I would add to the causes mentioned above, a pathological spasmodic retraction of the muscular tissue, *resulting from a perverted or disturbed condition of the excito-motor apparatus of the medulla spinalis; especially of that portion which is in direct relation with the nervous branches distributed among the pelvi-femoral muscles.*"

We cannot spare any more space for extended quotations, and must be content with quoting the author's views of the cause of the muscular spasmodic retraction, upon which these dislocations depend. Dr. Carnochan says this cause is "*a perverted condition of the excito-motor*

*apparatus of the medulla spinalis, and more especially of that portion of it which is in direct relation with the reflex-motor nervous fibres, distributed to the pelvi-femoral muscles surrounding, and in connexion with, the ilio-femoral articulation.*"

On the subject of the successful treatment of congenital dislocations of the femur we have but little faith, although a commission of the Royal Academy of Medicine, composed of MM. Blandin, Gerdy, Sanson, and Naquart, reported, in 1838, favorably upon the cases of M. Pravaz. We think less favorably of the matter, perhaps, on account of M. Guérin's connection with it. His orthopedic affairs have always seemed to us to border on quackery. But a commission, nominated by the Council General of the Civil Hospitals of Paris, consisting of MM. Rayer, Serres, Louis, Jobert, Blandin, and Orfila, reported favorably on the peculiar treatment adopted by M. Guérin. In those cases "where the head of the femur has been reduced, and where, from the continued muscular retraction, and the defective formation of the acetabulum, it has been found impossible to retain the head of the femur in its normal position, M. Guérin has advised the division of the retracted muscles, and has also practiced subcutaneous scarifications in the vicinity of the cotyloid cavity, in order to provoke an effusion of organizing material, by which the defect in the acetabulum may be remedied, and the head of the femur thus be retained in its cavity, and supplied with a sufficient *point d'appui.*"

This work is devoted to an attempt at extending the powers of surgery, and as such is entitled to the attention of the profession. We are not confident of the success of the measures commended, but they deserve to be tried. And we have already said that a knowledge of the contents of this book may save a physician from disagreeable blunders. It is important to distinguish congenital dislocation, from hip-joint disease, and a clear method of diagnosis is marked out in the fifth chapter of this work. M. Guérin has described, also, a class of congenital deformities at the hip which he has named pseudo-luxations. In these the head of the femur is not transferred from the cotyloid cavity. There are two varieties of these pseudo-luxations, one simulating a dislocation of the femur backwards and outwards; the other, a dislocation of the bone downwards and forwards.

"There are certain alterations in the head and neck of the thigh bone, and of the acetabulum, which may also give rise to some doubt in forming the diagnosis of this congenital dislocation. We find that Palleta speaks of several malformations, which may resemble this affection; such as an unusual enlargement of the acetabulum, permitting a vacillating motion of the head of the femur; a malformation of the upper extremity of the femur, where the head and neck are in part atrophied, and, as it were, fused with the trocanter major which rises above them; an exostosis springing from the bottom of the acetabulum, and changing the natural relations of the femur; and an atrophy, manifesting itself upon one half of the pelvis and upon the inferior extremity of the same side.

"Mr. Liston mentions a curious case of malady of the hip joint,* of a young infantry soldier, who died of pulmonary consumption after two years' confinement in the General Hospital at Chatham. In this case, the head of the bone was found approximated to the shaft, owing to absorption of the neck, without any ulceration of the articular cartilage. 'Previously to his admission, he had regularly performed his duty, from which it is plain that his limbs were then of equal length, although when his body was examined, the affected femur was *upwards of one inch and a half shorter than the other.* From a care-

---

*Liston's Surgery—report of a case of Mr. Gulliver's.

ful inquiry, after his death, it appeared that he had, five years previously, fallen on the trochanter, in consequence of which he often complained of pain in the hip, but continued to do his duty long after, never having been confined on account of the accident."* The shortening of the limb in this case is attributed to the accident, and to the long confinement with the pulmonary malady, which ultimately proved fatal.

Sir Benjamin Brodie has noticed that the lower extremities are sometimes of unequal length, from original conformation; the femur, tibia and fibula of one side being shorter than these bones of the other side.

In aged persons, the neck of the femur becomes sometimes altered by interstitial absorption, causing an unnatural shortening of the inferior extremities; and this circumstance predisposes in a remarkable degree to fracture of the neck of the femur, accompanied by still farther shortening of the limb.

Delpech† has mentioned an osseous malformation causing lameness, most probably depending on a rachitic condition, where there is malposition of the acetabula either more forwards or more backwards than natural. The same surgeon also mentions an atrophied state of the halves of the sacrum and of the ilium, as causing lameness; and M. Gerdy has described several anomalies of the upper extremity of the femur, from which a limping gait is produced, where the neck of this bone is inserted at a greater or less distance from the trochanter major, and in a direction, sometimes inclining obliqnely forwards; at others, obliquely backwards.

"All these morbid affections may simnlate, more or less, the congenital luxation of the head of the femur. A

---

*Liston's Surgery—report of a case of Mr. Gulliver's.

†Delpech l'Orthomorphie.

knowledge of, and familiarity with their existence, and the application to them of the signs which have been enumerated and described for determining the pathognomonic characteristics which accompany congenital dislocation of the femur upon the ilium, will enable the surgeon to arrive with great certainty at a just differential diagnosis."

We think the author of this work is entitled to the thanks of the profession for this interesting essay. It is well written, and thirteen plates admirably illustrate the doctrines of the text. The work is published in very creditable style.

This work may be obtained at the book-store of Messrs. Morton & Griswold.

